# Gene expression pattern of the epidermal growth factor receptor family and LRIG1 in renal cell carcinoma

**DOI:** 10.1186/1756-0500-5-216

**Published:** 2012-05-03

**Authors:** Marcus Thomasson, Håkan Hedman, Börje Ljungberg, Roger Henriksson

**Affiliations:** 1 Department of Radiation Sciences, Umeå University, SE-901 87, Umeå, Sweden; 2 Department of Surgery and Perioperative Sciences, Urology and Andrology, Umeå University, SE-901 87, Umeå, Sweden

**Keywords:** Renal cell carcinoma, EGFR, ERBB2, ERBB3, ERRB4, LRIG1, Survival

## Abstract

**Background:**

Previous studies have revealed altered expression of epidermal growth factor receptor (EGFR)-family members and their endogenous inhibitor leucine-rich and immunoglobulin-like domains 1 (LRIG1) in renal cell carcinoma (RCC). In this study, we analyzed the gene expression levels of EGFR-family members and LRIG1, and their possible associations with clinical parameters in various types of RCC.

**Methods:**

Gene expression levels of EGFR–family members and LRIG1 were analyzed in 104 RCC samples, including 81 clear cell RCC (ccRCC), 15 papillary RCC (pRCC), and 7 chromophobe RCC (chRCC) by quantitative real-time RT-PCR. Associations between gene expression levels and clinical data, including tumor grade, stage, and patient survival were statistically assessed.

**Results:**

Compared to kidney cortex, *EGFR* was up-regulated in ccRCC and pRCC, *LRIG1* and *ERBB2* were down-regulated in ccRCC, and *ERBB4* was strongly down-regulated in all RCC types. *ERBB3* expression did not differ between RCC types or between RCC and the kidney cortex. The expression of the analyzed genes did not correlate with patient outcome.

**Conclusions:**

This study revealed that the previously described up-regulation of *EGFR* and down-regulation of *ERBB4* occurred in all analyzed RCC types, whereas down-regulation of *ERBB2* and *LRIG1* was only present in ccRCC. These observations illustrate the need to evaluate the different RCC types individually when analyzing molecules of interest and potential biological markers.

## Findings

### Background

Renal cell carcinoma (RCC) consists of various tumor types [1]; clear cell RCC (ccRCC) accounts for approximately 70-80 % of the RCCs, papillary RCC (pRCC) for 10-15 % of cases, chromophobe RCC (chRCC) for approximately 5 %, and collecting duct carcinoma for less than 1 % of RCCs. Approximately 4-5 % of RCCs do not fit the histopathological criteria and are referred to as unclassified carcinomas [1]. The RCC types represent tumor groups with different genetic and molecular properties, as reviewed in [2] and [3]. When RCC types are analyzed collectively, the results predominantly reflect the properties of ccRCC, since this type accounts for the majority of RCC cases. Previous studies have revealed altered expression of epidermal growth factor (EGF) receptor (EGFR)-family members and their endogenous inhibitor leucine-rich and immunoglobulin-like domains 1 (LRIG1) in renal cell carcinoma (RCC). The EGFR family consists of four receptor tyrosine kinases, EGFR (ERBB1, HER1), ERBB2 (HER2, neu), ERBB3 (HER3), and ERBB4 (HER4) [4], of which down-stream intracellular signaling pathways regulate cell proliferation, differentiation, and migration [5]. LRIG1 [6] negatively regulates all four members of the EGFR-family [7-10]. LRIG1 is also a negative regulator of the MET - and RET -receptor tyrosine kinases [11,12]. LRIG1 is down-regulated in several cancers and cancer cell lines, including breast cancer and squamous cell carcinoma of the skin and uterine cervix, where low LRIG1 expression correlates with poor patient survival [13-17].

Previously, we reported the expression of the EGFR-family members and LRIG1 in a limited number of RCC-patients [18,19]. Here, we extended these studies to a larger patient cohort, and analyzed the RCC types individually.

## Methods

In this study we analyzed tumor samples from 104 patients who underwent nephrectomy at the Department of Urology, Umeå University Hospital, between the years 1986 and 1999 (Table 1). These tumors included 81 ccRCC, 15 pRCC, 7 chRCC, and 1 unclassified carcinoma. Additionally, specimens of histologically verified non-neoplastic kidney cortex were obtained from 27 of the nephrectomized kidneys. RNA was prepared and quantitative real-time reverse transcription- (RT-) PCR of *EGFRERBB2, ERBB3, ERBB4LRIG1,* and RN18S1 (18 S rRNA) was performed as previously described [18,19]. To correct for differences in RNA quality and quantity, apparent levels of RN18S1 were used to normalize the *EGFRERBB2-4,* and *LRIG1* values in each respective RNA sample. To test the reliability of the analysis, all five protein encoding genes were analyzed five times for three different samples. The maximum coefficient of variation and the standard deviation, expressed as a percentage of the mean, was 22 %. Patients provided informed consent for the use of both their tumor material and clinical data for studies. This study was approved by the research ethics committee at Umeå University Medical Faculty (No 02–340).

**Table 1:** Characteristics of the patients and tumors included in the study

**Total no. of patients**		**104**
Sex	male/female	56/48
Age in years	median (range)	65 (25–85)
Tumor diameter in mm	median (range)	80 (30–250)
Survival in months	Range	0-130
Tumor stage (WHO)	I	26
(As derived from TNM)	II	15
	III	30
	IV	33
Tumor grade (Fuhrman)	1	4
	2	14
	3	60
	4	26
RCC type by histology	Clear cell	81
	Papillary	15
	Chromophobe	7
	Unclassified	1
Patients with known metastasis at diagnosis		35
Patients who died from the disease		57
Patients dead from other causes		16
Patients alive at last follow-up (with disease)		31 (3)

Statistical analysis was performed using nonparametric statistics, as normal distribution of the data could not be assumed. For comparisons between two groups, the Mann–Whitney U-test was used. Comparisons of more than two groups were performed using the Kruskal-Wallis test. Comparisons between coupled samples were performed using the Wilcoxon signed-rank test. Correlations were analyzed according to Spearman’s rank correlation. Survival analysis was performed by first comparing patients with mRNA expression levels either above or below the median and then subjecting the data to Kaplan-Meier analysis by log-rank test. All P-values were two-sided. All calculations were performed using SPSS 14.0 software.

## Results

**Figure 1:**
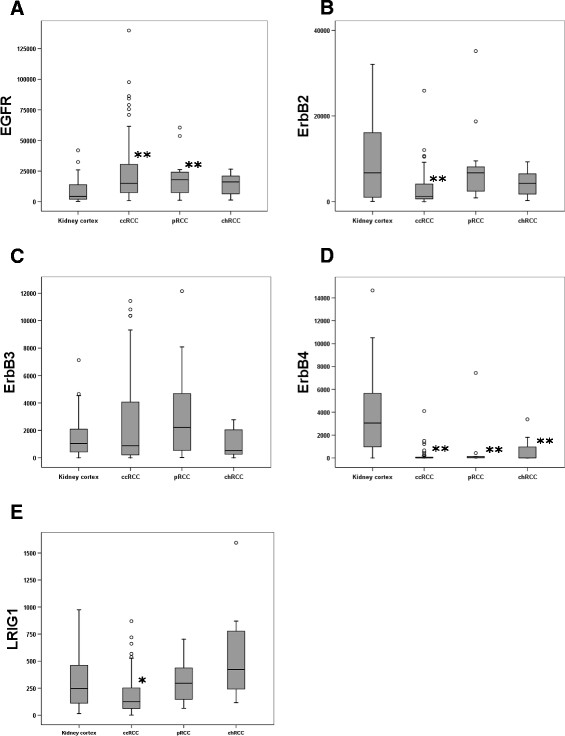
**Boxplots of the relative mRNA expression of the*EGFR*-family members and*LRIG1*in both the kidney cortex and the RCC types.** Relative mRNA expression of *EGFR*, *ERBB2, ERRB3, ERRB4,* and *LRIG1* was quantified in kidney cortex (n = 27), ccRCC (n = 81), pRCC (n = 15), and chRCC (n = 7). (A) *EGFR* mRNA expression was elevated in ccRCC and pRCC compared to kidney cortex. (Increased expression in chRCC was not significant, but expression levels were similar to other RCC groups.) (B) *ERBB2* mRNA expression was significantly lower in ccRCC compared to kidney cortex. In pRCC and chRCC, expression did not significantly differ from kidney cortex. (C) *ERBB3* mRNA expression was not significantly different between any of the RCC types compared to the kidney cortex. (D) *ERBB4* mRNA expression was significantly reduced in all RCC types compared to kidney cortex. (E) *LRIG1* mRNA expression was significantly lower in ccRCC compared to kidney cortex. In pRCC and chRCC, LRIG1 expression did not significantly differ from kidney cortex. Outlier values are marked °. Significant differences compared to expression in the kidney cortex are labeled (*) for P < 0.05 and (**) for P < 0.01.

The expression of *EGFR* was higher in all RCC types combined compared to kidney cortex tissue (P < 0.001) (Table 2). This up-regulation was significant for both ccRCC (P < 0.001) and pRCC (P = 0.016), but not for chRCC (P = 0.257) (Figure 1A; Table 2). This is in line with earlier findings by us and others [18,20-24].

**Table 2:** Significant differences in mRNA expression between kidney cortex and the RCC types

	All RCC combined*	ccRCC*	pRCC*	chRCC*	**Altered expression**	**Difference RCC types†**
EGFR	< 0.001	0.001	0.016	NS	Up-regulation	NS
ErbB2	0.003	0.001	NS	NS	Down-regulation	< 0.001
ErbB3	NS	NS	NS	NS	-	NS
ErbB4	0.001	< 0.001	0.001	0.03	Down-regulation	NS
LRIG1	NS	0.015	NS	NS	Down-regulation	0.002

* P-values were calculated in comparison to kidney cortex using the Mann–Whitney U-test.

† Comparison of the significant differences between the RCC types was performed using the Kruskal-Wallis test.

NS, non-significant.

*ERBB2* expression was significantly reduced in all RCC types combined compared to kidney cortex (P = 0.003); however, reduced expression was only significant in ccRCC (P = 0.001) and not in pRCC or chRCC (Figure 1B). These results indicate that the previously described down-regulation of *ERBB2* in RCC [19] is actually a result of down-regulation in the ccRCCs.

*ERBB3* expression was similar in RCC and non-neoplastic kidney cortex, and there was no significant expression difference between RCC types (Figure 1C).

*ERBB4* expression was markedly lower in all the different RCC types than in kidney cortex (P < 0.001). There was no difference in *ERBB4* expression between the RCC types (Figure 1D). Thus, the earlier described down-regulation of *ERBB4* in RCC [19] was here shown to be prominent in all RCC types analyzed. In fact, no *ERBB4* expression could be detected in 51 out of the 104 tumors analyzed. This pronounced down-regulation of *ERBB4* may suggest an important role for this receptor tyrosine kinase in inhibiting the development of RCC.

The expression *of LRIG1* was reduced in ccRCC compared to kidney cortex (P = 0.020). The expression of *LRIG1* in pRCC and chRCC was not significantly different from that of kidney cortex (Figure 1E). Thus, the previously described down-regulation of LRIG1 in RCC [18] was restricted to ccRCC. This finding could indicate a tumor suppressive role for LRIG1 in the context of ccRCC that is not present or of reduced importance in other types of RCC.

The expression levels of *EGFRERBB2-4,* and *LRIG1* correlated significantly with each other in most cases (Table 3). This was possibly due to LRIG1 expression being up-regulated by receptor activation [7] or due to metholodical issues, e.g. due to variation in the expression of the reference gene, RN18S1. There was a non-significant trend to an inverse correlation (R = −0.166 P = 0.058) between the expression levels of *EGFR* and *ERBB4*. This results is in line with *EGFR* being up-regulated and *ERBB4* being down-regulated in tumors.

**Table 3:** Correlation of mRNA expression levels between the analyzed genes

		**EGFR**	**ErbB2**	**ErbB3**	**ErbB4**	**LRIG1**
EGFR	CC	1.000	0.274*	0.425*	−0.166	0,370*
	P-value	-	0.002	<0.001	0.058	<0.001
ErbB2	CC	0.274*	1.000	0.586*	0.375*	0.456*
	P-value	0.002	-	<0.001	<0.001	<0.001
ErbB3	CC	0.425*	0.586*	1.000	0.152	0.456
	P-value	<0.001	<0.001	-	0.082	0.000
ErbB4	CC	−0.166	0.375*	0.152	1.000	0.277*
	P-value	0.058	<0.001	0.082	-	0.001
LRIG1	CC	0.370*	0.456	0.456	0.277*	1.000
	P-value	<0.001	<0.001	<0.001	0.001	-

CC, Correlation coefficient according to Spearman.

*Significant with P-value <0.05.

The expression levels of *ERBB3* within ccRCC were inversely correlated with tumor grade and tumor size (R = −0.287, P = 0.009 and R = −0.244, P = 0.027, respectively). As expression of *ERBB3* mRNA was low and did not differ between tumors and kidney cortex or between RCC types, the biological significance of this finding is highly uncertain. No other significant correlation was observed between the expression of *EGFR*-family members or *LRIG1* and the size, grade, or stage of the tumors. Survival analysis comparing patients with tumors expressing above or below median mRNA values of the five genes revealed no significant difference in overall survival or cancer specific survival, neither in ccRCC patients or all RCC patients combined ( Additional file 1 Figure S1). The patient groups for the other RCC types were too small for meaningful survival analyses. The previously described and non-significant association between LRIG1 expression and tumor grade and patient survival [18] was not confirmed in the present and larger study. Therefore, it appears that although LRIG1 may possess a tumor suppressive function in ccRCC, it does not appear to be an important prognostic factor in RCC.

## Conclusions

This study demonstrated that the expression of *EGFR*, *ERBB3* and *ERBB4* was similar throughout different RCC types, whereas the expression of *ERBB2* and *LRIG1* differed between the various types of RCC. This demonstrates potentially important differences and similarities in the expression of the EGFR-family members and LRIG1 genes between different RCC types. Up-regulated gene expression of *EGFR* compared to kidney cortex was found in all RCC types analyzed. A strong down-regulation of *ERBB4* was observed in all RCC types analyzed, while down-regulation of *ERBB2* and *LRIG1* was found only in ccRCC. The biological and clinical significance of these differences in gene expression warrants further study.

## Abbreviations

EGFR = Epidermal growth factor receptor; ERBB = from avian erythroblastosis oncogene B, gene encoding for EGFR; HER = Human EGF receptor; Neu = old designation given to ERRB2 gene when first found in neural mouse tumors; LRIG1 = Leucine-rich repeats and immunoglobulin-like domains-1; MET = Gene encoding the hepatocyte growth factor receptor; RCC = Renal cell carcinoma; ccRCC = Clear cell (also called conventional) RCC; chRCC = Chromophobe RCC; pRCC = Papillary RCC; RET = A receptor tyrosine kinase, receptor for members of the glial cell line-derived neurothrophic factor (GDNF) family.

## Competing interests

The authors declare that they have no competing interests.

## Author’s contribution

MT performed RT-PCR analysis, statistical analysis, and drafted the manuscript. HH provided support for the RT-PCR analyses and helped draft the manuscript. BL supervised collection of tumor samples, RNA extracts and clinical data. RH coordinated the study. All authors participated in the conception and design of the study. All authors have read and approved the final manuscript.

## Supplementary Material

Additional file 1 **Figure S1**Kaplan-Meier survival curves of cancer specific survival comparing patients with tumors above and below median expression of all five genes. Click here for file

## References

[B1] Störkel S Eble J Adlakha K Amin M Blute M Bostwick D Darson M Delahunt B Iczkowski K Classification of renal cell carcinoma: Workgroup No. 1. Union Internationale Contre le Cancer (UICC) and the American Joint Committee on Cancer (AJCC) Cancer 1997 80 987 989 10.1002/(SICI)1097-0142(19970901)80:5<987::AID-CNCR24>3.0.CO;2-R 9307203

[B2] Linehan WM Vasselli J Srinivasan R Walther MM Merino M Choyke P Vocke C Schmidt L Isaacs JS Glenn G Genetic basis of cancer of the kidney: disease-specific approaches to therapy Clin Cancer Res 2004 10 6282S 6289S 10.1158/1078-0432.CCR-050013 15448018

[B3] Baldewijns MM van Vlodrop IJ Schouten LJ Soetekouw PM de Bruine AP van Engeland M Genetics and epigenetics of renal cell cancer Biochim Biophys Acta 2008 1785 133 155 18187049 10.1016/j.bbcan.2007.12.002

[B4] Yarden Y Sliwkowski M Untangling the ErbB signalling network Nat Rev Mol Cell Biol 2001 2 127 137 10.1038/35052073 11252954

[B5] Olayioye M Neve R Lane H Hynes N The ErbB signaling network: receptor heterodimerization in development and cancer EMBO J 2000 19 3159 3167 10.1093/emboj/19.13.3159 10880430 PMC313958

[B6] Nilsson J Vallbo C Guo D Golovleva I Hallberg B Henriksson R Hedman H Cloning, characterization, and expression of human LIG1 Biochem Biophys Res Commun 2001 284 1155 1161 10.1006/bbrc.2001.5092 11414704

[B7] Gur G Rubin C Katz M Amit I Citri A Nilsson J Amariglio N Henriksson R Rechavi G Hedman H LRIG1 restricts growth factor signaling by enhancing receptor ubiquitylation and degradation EMBO J 2004 23 3270 3281 10.1038/sj.emboj.7600342 15282549 PMC514515

[B8] Laederich MB Funes-Duran M Yen L Ingalla E Wu X Carraway KL Sweeney C The leucine-rich repeat protein LRIG1 is a negative regulator of ErbB family receptor tyrosine kinases J Biol Chem 2004 279 47050 47056 10.1074/jbc.M409703200 15345710

[B9] Goldoni S Iozzo RA Kay P Campbell S McQuillan A Agnew C Zhu JX Keene DR Reed CC Iozzo RV A soluble ectodomain of LRIG1 inhibits cancer cell growth by attenuating basal and ligand-dependent EGFR activity Oncogene 2007 26 368 381 10.1038/sj.onc.1209803 16847455

[B10] Yi W Holmlund C Nilsson J Inui S Lei T Itami S Henriksson R Hedman H Paracrine regulation of growth factor signaling by shed leucine-rich repeats and immunoglobulin-like domains 1 Exp Cell Res 2011 317 504 512 10.1016/j.yexcr.2010.11.005 21087604

[B11] Shattuck DL Miller JK Laederich M Funes M Petersen H Carraway KL Sweeney C LRIG1 is a novel negative regulator of the Met receptor and opposes Met and Her2 synergy Mol Cell Biol 2007 27 1934 1946 10.1128/MCB.00757-06 17178829 PMC1820466

[B12] Ledda F Bieraugel O Fard SS Vilar M Paratcha G Lrig1 is an endogenous inhibitor of Ret receptor tyrosine kinase activation, downstream signaling, and biological responses to GDNF J Neurosci 2008 28 39 49 10.1523/JNEUROSCI.2196-07.2008 18171921 PMC6671136

[B13] Miller JK Shattuck DL Ingalla EQ Yen L Borowsky AD Young LJ Cardiff RD Carraway KL Sweeney C Suppression of the negative regulator LRIG1 contributes to ErbB2 overexpression in breast cancer Cancer Res 2008 68 8286 8294 10.1158/0008-5472.CAN-07-6316 18922900 PMC2597648

[B14] Hedman H Nilsson J Guo D Henriksson R Is LRIG1 a tumour suppressor gene at chromosome 3p14.3? Acta Oncol 2002 41 352 354 10.1080/028418602760169398 12234026

[B15] Tanemura A Nagasawa T Inui S Itami S LRIG-1 provides a novel prognostic predictor in squamous cell carcinoma of the skin: immunohistochemical analysis for 38 cases Dermatol Surg 2005 31 423 430 15871317 10.1111/j.1524-4725.2005.31108

[B16] Lindstrom AK Ekman K Stendahl U Tot T Henriksson R Hedman H Hellberg D LRIG1 and squamous epithelial uterine cervical cancer: correlation to prognosis, other tumor markers, sex steroid hormones, and smoking Int J Gynecol Cancer 2008 18 312 317 10.1111/j.1525-1438.2007.01021.x 17624990

[B17] Krig SR Frietze S Simion C Miller JK Fry WH Rafidi H Kotelawala L Qi L Griffith OL Gray JW Lrig1 Is an Estrogen-Regulated Growth Suppressor and Correlates with Longer Relapse-Free Survival in ER{alpha}-Positive Breast Cancer Mol Cancer Res 2011 9 1406 1417 10.1158/1541-7786.MCR-11-0227 21821674 PMC3196675

[B18] Thomasson M Hedman H Guo D Ljungberg B Henriksson R LRIG1 and epidermal growth factor receptor in renal cell carcinoma: A quantitative RT-PCR and immunohistochemical analysis Br J Cancer 2003 89 1285 1289 10.1038/sj.bjc.6601208 14520461 PMC2394322

[B19] Thomasson M Hedman H Junttila TT Elenius K Ljungberg B Henriksson R ErbB4 is downregulated in renal cell carcinoma–a quantitative RT-PCR and immunohistochemical analysis of the epidermal growth factor receptor family Acta Oncol 2004 43 453 459 10.1080/02841860410028574 15360049

[B20] Hofmockel G Riess S Bassukas I Dammrich J Epidermal growth factor family and renal cell carcinoma: expression and prognostic impact Eur Urol 1997 31 478 484 9187911 10.1159/000474510

[B21] Yoshida K Tosaka A Epidermal growth factor binding by membranes of human renal cell carcinomas: establishment of an epidermal growth factor receptor assay for clinical use Int J Urol 1994 1 319 323 10.1111/j.1442-2042.1994.tb00057.x 7614394

[B22] Lager D Slagel D Palechek P The expression of epidermal growth factor receptor and transforming growth factor alpha in renal cell carcinoma Mod Pathol 1994 7 544 548 7937720

[B23] Ljungberg B Gafvels M Damber J Epidermal growth factor receptor gene expression and binding capacity in renal cell carcinoma, in relation to tumor stage, grade and DNA ploidy Urol Res 1994 22 305 308 10.1007/BF00297200 7879316

[B24] Sakaeda T Okamura N Gotoh A Shirakawa T Terao S Morioka M Tokui K Tanaka H Nakamura T Yagi M EGFR mRNA is upregulated, but somatic mutations of the gene are hardly found in renal cell carcinoma in Japanese patients Pharm Res 2005 22 1757 1761 10.1007/s11095-005-7094-2 16180134

