# Supplementary material for: Gene expression pattern of the epidermal growth factor receptor family and LRIG1 in renal cell carcinoma
Source: BMC Res Notes. 2012 May 3;5:216. doi: 10.1186/1756-0500-5-216 (PMC3419632; doi:10.1186/1756-0500-5-216)
Supplement: Additional file 1 — Figure S1Kaplan-Meier survival curves of cancer specific survival comparing patients with tumors above and below median expression of all five genes. [file 1756-0500-5-216-S1.pdf]

Supporting Figure 1

A

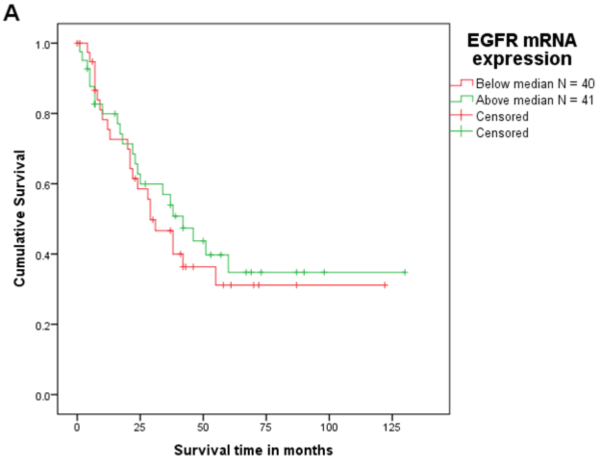

B

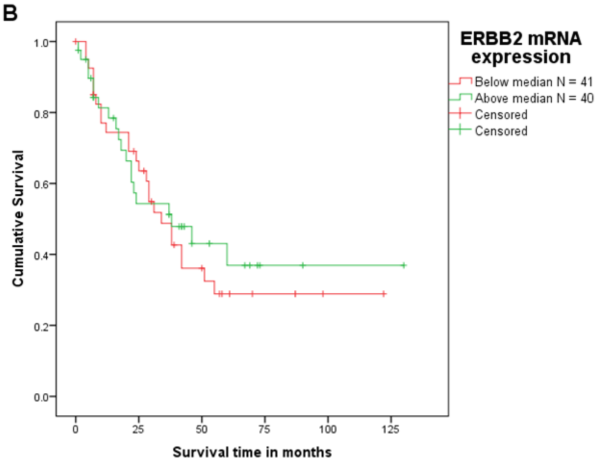

C

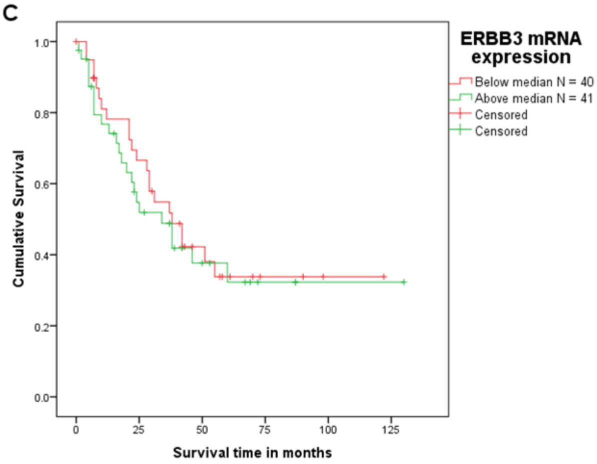

D

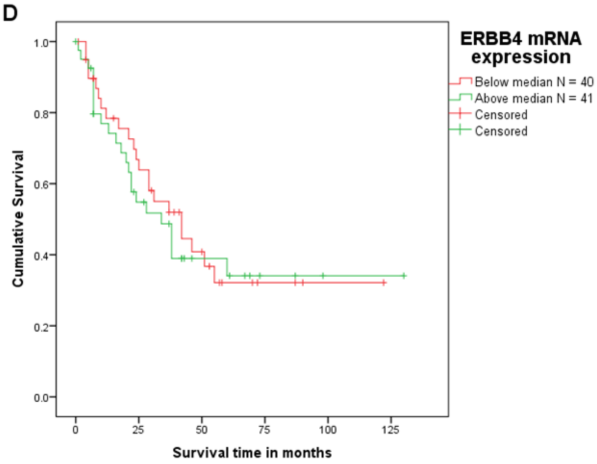

E

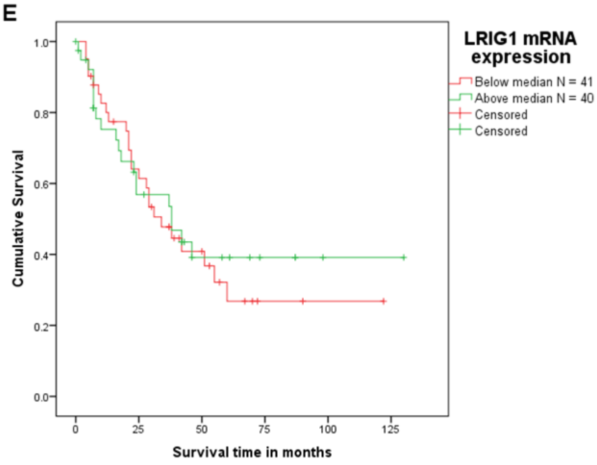

Cancer specific survival for ccRCC patients with tumors expressing above and below the median gene expression levels of the *EGFR*-family members and *LRIG1*, respectively.

Kaplan-Meier plots of the 81 patients with ccRCC in the study, of whom 48 died from the disease. Patients who died from other reasons or was alive at the time of last follow-up were censored. Patient samples were analyzed for expression of *EGFR* (A), *ERBB2* (B), *ERBB3* (C), *ERBB4* (D), and *LRIG1* (E). For each gene, the patients were separated into two groups, those with tumors expressing mRNA levels above and below the median, respectively. P-values were calculated according to the log-rank test: *EGFR* (P = 0.620), *ERBB2* (P = 0.691), *ERBB3* (P = 0.624), *ERBB4* (P = 0.731), and *LRIG1* (P = 0.733).

Analysis with similar methodology for overall survival in the ccRCC patient group and overall, and cancer specific, survival in all RCC patients combined were also performed. No significant difference in survival was found in these analyses (data not shown).
